# Deodorization of Recycled HDPE: Comparative Assessment of Washing and Solvent-Based Purification Strategies with a Techno-Economic Analysis

**DOI:** 10.3390/polym18121441

**Published:** 2026-06-09

**Authors:** Aymara Blanco, Vafa Feyzi, Rafael Juan, Beatriz Paredes, Carlos Domínguez, Javier Dufour, Rafael A. García-Muñoz

**Affiliations:** 1 Polymer Technology Laboratory (LATEP), Rey Juan Carlos University, Tulipán St., 28933 Móstoles, Madrid, Spain; aymara.blanco@urjc.es (A.B.); rafael.juan@urjc.es (R.J.); beatriz.paredes@urjc.es (B.P.); carlos.domiguez@urjc.es (C.D.); 2 GIQA, Group of Environmental and Chemical Engineering, ESCET, Rey Juan Carlos University, Tulipán St., 28933 Móstoles, Madrid, Spain; javier.dufour@imdea.org; 3 Systems Analysis Unit, IMDEA Energy, Avda. Ramón de la Sagra 3, 28935 Móstoles, Madrid, Spain; vafa.feyzi@imdea.org; 4 Instituto de Investigación de Tecnologías para la Sostenibilidad, Rey Juan Carlos University, Tulipán St., 28933 Móstoles, Madrid, Spain

**Keywords:** recycled HDPE, VOCs removal, NIASs, ultrasound-assisted washing, techno-economic analysis, circular economy

## Abstract

Residual volatile organic compounds (VOCs) and non-intentionally added substances (NIASs) limit the reuse of post-consumer recycled high-density polyethylene (rHDPE) in high-value applications because they generate persistent odors and may compromise product quality and regulatory acceptance. This work comparatively assesses five deodorization and purification routes for rHDPE: agitation washing, ultrasound-assisted washing, reflux heating, Soxhlet extraction, and dissolution/precipitation, by combining VOC removal performance, material characterization, and techno-economic evaluation. Ultrasound-assisted washing with SDS achieved ~96% total VOC removal, while reflux heating resulted in near-complete removal (~98%), approaching the analytical detection limit. Soxhlet extraction with ethanol reached 94% after 1 h, and the dissolution/precipitation method provided near-complete purification and removed additional impurities, but at the expense of substantially higher process complexity and cost. Mechanical and physicochemical characterization indicated that the evaluated treatments did not appreciably compromise the measured properties of the recycled polymer. In addition, equilibrium screening with representative analytes in ethanol provided qualitative support for the solvent–polymer interaction discussion. A plant-scale techno-economic assessment identified ultrasound-assisted SDS washing as the most attractive option, offering the best balance between deodorization efficiency, process simplicity, and cost. Overall, the results provide a practical basis for selecting scalable decontamination strategies to upgrade rHDPE quality and expand its use in circular plastic applications.

## 1. Introduction

Plastics recycling in the European Union has shown a positive evolution in recent years, with post-consumer recycling rates reaching 39.8% in 2023, reflecting increased commitment to circular economy strategies and plastic waste management [1,2,3]. Despite this progress, the quality of recycled polymers remains a key limiting factor for their use in higher-value applications.

One of the most widely used polymers in packaging applications is high-density polyethylene (HDPE), a polyolefin valued for its mechanical strength, chemical resistance, and cost-effectiveness. However, its porous structure and high surface area make it susceptible to the adsorption of volatile organic compounds (VOCs), which often persist after recycling and limit its reuse in high-value applications [4,5].

VOCs in recycled plastics originate from previous product contents, degradation reactions during processing, and residual additives, and are a major source of persistent off-odors and quality limitations [6,7]. Many are classified as non-intentionally added substances (NIASs), which can affect safety, acceptance, and regulatory compliance, particularly in food- or cosmetic-related applications [8,9,10].

Therefore, the accumulation of VOCs in recycled HDPE can generate residual odors and affect the quality of the material, and that implies the need for decontamination strategies [5,11]. To address this issue, various deodorization techniques have been explored. Mechanical recycling through extrusion can provide partial VOC removal when processing conditions are optimized, although unpleasant odors often persist [7,10]. More advanced approaches, such as zeolite compounding, have shown improved odor reduction without compromising mechanical properties, but face limitations related to additive costs, abrasive behavior, adsorption saturation, and waste management requirements [6,12].

Therefore, another considerable challenge in odor removal from recycled plastics is preserving the mechanical and thermal properties of the material during the deodorization process. Techniques such as hot air application and solvent extraction are effective in VOC removal but may induce polymer degradation if not carefully controlled, potentially generating new volatile contaminants and compromising material quality [13].

Simple washing techniques using water or detergents are easy to implement but typically remove only 50–65% of VOCs and perform poorly against apolar or strongly bound compounds due to their low water solubility and strong adhesion to the polymer matrix [14,15,16,17]. In contrast, more advanced techniques such as sonication and the use of organic solvents have shown greater effectiveness in removing VOCs. Sonication is especially effective in breaking down persistent compounds that traditional methods fail to remove completely, achieving removal efficiencies of up to 90% in some studies [18,19]. On the other hand, the application of organic solvents, such as ethyl acetate or surfactants dissolved in water, such as hexadecyl trimethyl ammonium (CTAB), can dissolve a broad range of organic compounds, including those of apolar character, with removal efficiencies as high as 95% in certain cases [10].

The advanced dissolution/precipitation process has shown great potential in the deodorization of recycled plastics. This process involves dissolving the plastic in an appropriate solvent and then precipitating it, thereby removing the volatile contaminants along with the solvent. According to other research, the use of environmentally friendly solvents in temperature-induced, anti-solvent phase separation not only reduces odors but also improves the purity and quality of the recycled plastic by additionally removing inorganic additives [20,21]. Despite its advantages (high removal efficiency, improved material quality, and environmental compatibility), challenges related to solvent recovery, process cost, and scalability remain [22].

Efficient removal of VOCs is therefore essential to improve the quality and applicability of recycled HDPE while preserving its mechanical and thermal integrity and ensuring feasibility at an industrial scale. Despite extensive experimental research on deodorization techniques, the transition to large-scale implementation remains limited by the lack of comparative techno-economic assessments.

In this context, the present study systematically evaluates five deodorization and purification routes for post-consumer rHDPE: agitation washing, ultrasonic washing, washing reflux heating, Soxhlet extraction, and an advanced dissolution/precipitation process, while also integrating experimental performance with a screening-level techno-economic analysis.

Although several deodorization and purification approaches have been individually reported for recycled polyolefins, direct comparisons under consistent experimental conditions, including both material performance and techno-economic feasibility, remain limited. The novelty of this work lies in the integrated comparison of five VOC-removal strategies for post-consumer rHDPE, combining VOC removal efficiency, physicochemical and mechanical characterization, and screening-level techno-economic analysis. This approach enables the identification of scalable alternatives that balance deodorization performance, polymer integrity, process complexity, and cost.

## 2. Experimental Part

### 2.1. Materials

The recycled high-density polyethylene (rHDPE) used in this study was sourced from post-consumer packaging waste, including bottles for detergents, cosmetics, and food. The material was supplied by Cordoplas (Córdoba, Spain), a Spanish recycling company.

Washing media included distilled water, ethanol (EtOH), ethyl acetate (AcOEt), acetone, carbon disulfide (CS_2_), cetyltrimethylammonium bromide (CTAB), sodium dodecyl sulfate (SDS), and sodium dodecylbenzene sulfonate (SDBS), all of them supplied by Sigma-Aldrich (Tokyo, Japan) with >99% purity. For dissolution/precipitation, ortho-dichlorobenzene (oDCB) was used as a solvent and acetone as a precipitating agent.

### 2.2. Instrumentation

Sample preparation and washing procedures were carried out using standard laboratory equipment: analytical balance (Mettler Toledo, Tokyo, Japan), magnetic stirring hot plate (IKA), and ultrasonic bath.

### 2.3. Methodology

**Figure 1:**
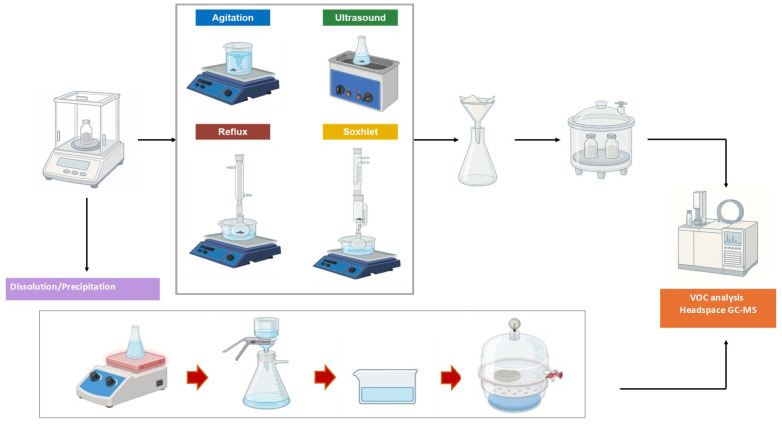
Schematic of the experimental procedure for the removal of VOCs in recycled PE by agitation, ultrasound, reflux heating, Soxhlet extraction, and dissolution/precipitation processes.

For each washing method, 5 g of rHDPE were contacted with 100 mL of solvent or surfactant solution; SDS, SDBS, and CTAB were used at 0.32% *w*/*v*. After treatment, samples were filtered, rinsed with distilled water, and dried before characterization (Figure 1).

Four washing configurations were evaluated under comparable conditions: (i) agitation washing at room temperature using magnetic stirring (200 rpm, 1 h); (ii) ultrasound-assisted washing at room temperature (1 h); (iii) reflux-assisted washing at controlled temperature (43 or 60 °C, 1 h); and (iv) Soxhlet extraction using continuous solvent circulation (1 h).

For dissolution/precipitation, 1 g of rHDPE was dissolved in 192 mL of oDCB (0.4% *w*/*w*) at 160 °C for 1 h, vacuum-filtered, precipitated with acetone (1:2 *v*/*v*), and dried for characterization (Figure 1 and Figure S1).

VOC analysis was performed using a headspace gas chromatography–mass spectrometry system (Bruker GC 450). Sample volatiles were allowed to accumulate in the headspace of sealed vials. An aliquot (1 mL) of headspace vapor was manually injected into the GC-MS system. Prior to injection, the sampling needle was preheated at 65 °C for 10 min in a CG oven to minimize condensation of volatile compounds.

The GC-MS system was equipped with a Br-5 ms capillary column (5% diphenyl/95% dimethyl polysiloxane, 30 m × 0.25 mm i.d., 0.25 µm film thickness). The injection port was maintained at 280 °C and operated in splitless mode. All experiments were carried out in triplicate. VOC removal efficiencies exceeding 99% correspond to residual concentrations below the detection limit of the GC–MS and are therefore discussed as near-complete removal rather than absolute quantitative values.

For the measurements of apparent polymer–ethanol distribution coefficients, K^app^_p/w_, four chromatography vials were prepared, each containing 1 mL of ethanol spiked with 20 ppm of toluene, hexane, and limonene, as well as 1000 ppm of ethylenediamine. Six pellets of virgin polyethylene (PE) were added to each vial. The vials were then placed under continuous agitation for 72 h at room temperature. After this exposure period, the solutions were measured again. Quantitative analyses at time zero and after 72 h in contact with PE were performed by GC–MS/MS using a Bruker 320-MS GC Triple Quadrupole Mass Spectrometer (Bruker Corporation, Bremen, Germany), equipped with an SC-Wax capillary column (30 m × 0.25 mm, 0.25 μm film thickness). A 3 μL aliquot of each sample was injected for analysis.

## 3. Results and Discussion

### 3.1. Volatile Organic Compounds (VOCs) in Recycled Polyethylene

The post-consumer HDPE used in this study (recycled PE) comes from packaging applications such as cosmetic, detergent, and food containers. Due to prior use and degradation during processing, these materials can retain a wide variety of volatile organic compounds (VOCs), both intentionally added substances (IASs) and non-intentionally added substances (NIASs) [23].

GC-MS analysis identified a total of 31 VOCs in the recycled PE samples (Table S1), reflecting the chemical complexity of post-consumer contamination. The VOC profile of rHDPE was dominated by low-molecular-weight aromatic compounds, followed by less volatile aliphatic hydrocarbons and minor fractions of nitrogen-containing compounds and terpenes. The wide distribution of retention times observed in the chromatograms reflects the diverse origin and physicochemical properties of contaminants present in post-consumer plastics (Figure S2).

For interpretative purposes, the identified VOCs were classified into four main chemical families (Figures S3 and S4): aromatic hydrocarbons, which dominated the profile (~76%); aliphatic hydrocarbons (~13%); nitrogen-containing compounds, mainly amines (~8%); and terpenes (~2%). Aromatic hydrocarbons and amines are particularly relevant due to their strong odor contribution and potential impact on material acceptance, whereas terpenes, despite their low abundance, exhibit very low odor thresholds and can significantly influence perceived quality. These results highlight the complexity of VOC contamination in rHDPE and the need for targeted decontamination strategies that address both volatile and semi-volatile substances.

### 3.2. Evaluation of VOC Removal by Solvent-Based Washing Systems

VOC removal from recycled HDPE was evaluated using four solvent-based washing configurations, applying representative solvents and surfactants to target the main chemical families identified in the untreated material, namely aromatic and aliphatic hydrocarbons, amines, and terpenes.

#### 3.2.1. Washing at Room Temperature Under Agitation

**Figure 2:**
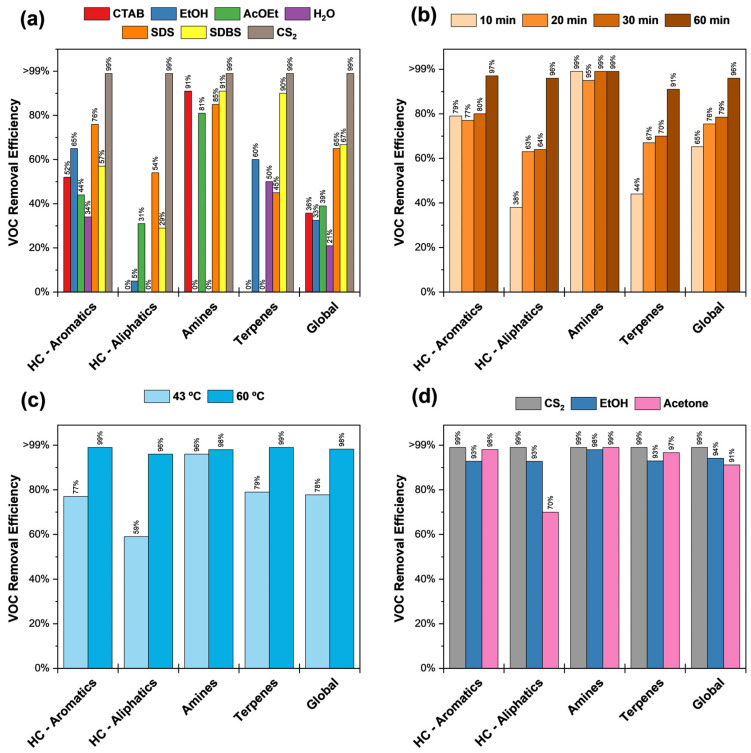
VOC removal efficiencies using (**a**) agitation washing system at room temperature; (**b**) ultrasonic washing system with SDS at different times; (**c**) reflux heating with SDS at 1 h, and (**d**) Soxhlet extractor system with different solvents. Removal values > 99% correspond to VOC concentrations below the detection limit of the GC–MS method and should be interpreted as near-complete removal rather than absolute quantification.

The agitation washing method at room temperature resulted in moderate VOC removal efficiencies, strongly dependent on solvent polarity and surfactant type (Figure 2a). Non-polar CS_2_ achieved near-complete VOC removal owing to its high affinity for hydrophobic compounds; however, due to its toxicity and handling constraints, it was used here primarily as a laboratory benchmark rather than as an industrially viable solvent [24].

In contrast, anionic surfactants such as SDS and SDBS demonstrated strong removal capabilities in aqueous media. SDS achieved 76% removal of aromatic hydrocarbons, 54% of aliphatics, and over 85% of amines, resulting in an overall efficiency of approximately 65%. These results can be attributed to the formation of micelles, supramolecular aggregates with a hydrophobic core, which encapsulate apolar VOCs and facilitate their solubilization in the aqueous phase. The SDS concentration used (0.32% *w*/*v*) exceeds its critical micelle concentration (CMC ≈ 8.2 × 10^−3^ mol/L), ensuring optimal micelle formation. SDBS yielded similar performance, especially for terpenes (90% removal) and amines (91%).

Cationic CTAB showed high selectivity for amines (91%), likely due to electrostatic interactions with nitrogen-containing groups. However, its overall efficiency remained lower (36%) due to the limited solubilization of aromatic and aliphatic compounds.

Among polar organic solvents, ethanol (EtOH) removed around 65% of aromatics, 33% overall, and was particularly effective against polar VOCs such as amines, while ethyl acetate (AcOEt) performed slightly better for aliphatics (31%).

To provide qualitative mechanistic support to the solvent-polymer interactions, preliminary equilibrium screening tests were carried out in ethanol using representative model compounds. These experiments were designed as screening measurements to estimate apparent polymer-wash distribution coefficients for selected analytes under sealed conditions. The values obtained show a clear contrast between the tested analytes (Supplementary Information S2, Table S2). Toluene, limonene, and ethylenediamine exhibited low K^app^_p/w_ values (0.087–0.259 mL·g^−1^), indicating limited uptake by PE under the selected conditions and suggesting that ethanol behaves as a favorable receiving phase for these compounds. In contrast, hexane showed a substantially higher K^app^_p/w_ value (4.06 mL·g^−1^), consistent with a greater tendency to partition into the polymer phase. These trends are qualitatively consistent with the washing results. In particular, the low K^app^_p/w_ obtained for ethylenediamine supports the good ability of ethanol to retain polar analytes in the liquid phase, whereas the larger K^app^_p/w_ obtained for hexane is in line with the lower effectiveness of ethanol toward aliphatic hydrocarbons compared with other organic media. The intermediate values obtained for toluene and limonene suggest that ethanol can also act as a reasonably effective sink for some aromatic and terpene compounds, although these single-analyte equilibrium tests should not be interpreted as direct predictors of the family-wise removal efficiencies observed for the complex VOC mixture present in real rHDPE.

However, none of these solvents approached the performance of CS_2_ or SDS-based systems. Regarding terpenes, represented by d-limonene, high removal was observed in CS_2_ (>99%) and SDBS (90%), while EtOH (60%) and SDS (54%) also performed acceptably. The amphiphilic nature of terpenes likely facilitates their encapsulation by micelles or solubilization in polar solvents [16,19].

In summary, agitation at room temperature using anionic surfactants, particularly SDS and SDBS, provides a cost-effective and environmentally safer alternative to nonpolar solvents like CS_2_, with acceptable removal efficiencies (>65%) for most VOC families. While less effective for tightly embedded or nonpolar VOCs, this method remains suitable for preliminary decontamination stages in recycling workflows [25].

#### 3.2.2. Removal of VOCs by Ultrasound-Assisted Washing

Ultrasound-assisted washing was evaluated as a means to enhance VOC removal through the combined action of SDS micelles and acoustic cavitation, which promotes VOC desorption and improves mass transfer into the washing medium [20]. Experiments were carried out with 0.32% (*w*/*v*) SDS for sonication times of 10, 20, 30, and 60 min (Figure 2b). VOC removal increased progressively with sonication time, confirming the beneficial effect of cavitation on extraction.

After 10 min, removal was already high for amines, which were almost completely eliminated, while aromatic hydrocarbons reached 79%, and aliphatics and terpenes showed lower values (58% and 44%, respectively). Extending sonication to 20 and 30 min mainly improved the removal of less polar compounds, particularly aliphatics and terpenes, whereas amines remained nearly fully removed [26,27]. After 60 min, near-complete removal was achieved for all groups: aromatics 97%, aliphatics 96%, amines 99%, and terpenes 91%, corresponding to an overall VOC removal of ~96%.

The time dependence suggests that ultrasound mainly accelerates desorption and facilitates micellar access to VOCs retained within the polymer matrix. Amines are removed rapidly because of their polarity and favorable interactions with the anionic surfactant, whereas aliphatics and terpenes require longer treatment due to their lower polarity and stronger retention in the polymer. Aromatics also show high removal, likely due to their efficient incorporation into the hydrophobic micellar core.

Overall, ultrasound-assisted washing with SDS is a highly effective deodorization route for recycled PE, combining high removal efficiency with moderate energy demand, and therefore represents a promising option for scalable decontamination.

#### 3.2.3. VOC Removal in Recycled PE by Reflux-Heated Washing

Reflux-assisted washing was evaluated as a thermal enhancement of the SDS-based process, maintaining continuous contact between the washing medium and rHDPE while preventing solvent loss. Two temperatures, 43 and 60 °C, were tested for 60 min (Figure 2c). Increasing temperature markedly improved VOCs removal, highlighting the role of thermal activation in the extraction process.

At 43 °C, total VOC removal reached 78%, with aromatic hydrocarbons, aliphatics, terpenes, and amines showing removals of 77%, 59%, 79%, and 96%, respectively. Raising the temperature to 60 °C increased total removal to ~98%, with values of 99% for aromatics, 96% for aliphatics, 99% for terpenes, and 98% for amines. These results represent the highest performance among the non-toxic surfactant-based systems evaluated.

The improvement with temperature can be attributed to enhanced VOC solubility in the washing medium, lower solution viscosity, and faster desorption from the polymer matrix. As in ultrasound-assisted washing, aromatics and amines showed high affinity for the SDS system, while aliphatics and terpenes benefited most from the temperature increase, likely because of their lower mobility and stronger retention in the polymer at milder conditions.

Overall, reflux-assisted washing with SDS provides near-complete VOC removal while preserving the advantages of aqueous surfactant systems. Its high efficiency and simple operation make it an attractive option for large-scale decontamination, although its final selection should be balanced against the lower energy demand of ultrasound-assisted washing by means of a comprehensive techno-economic analysis.

#### 3.2.4. Removal of VOCs in Recycled PE by Soxhlet Extraction Washing

Soxhlet extraction was evaluated as a continuous solvent-cycling technique that ensures prolonged contact between rHDPE and the washing medium under controlled thermal conditions, making it suitable for the removal of strongly retained VOCs [28,29]. Three solvents of different polarity were tested for 1 h: ethanol (EtOH), carbon disulfide (CS_2_), and acetone (Figure 2d).

CS_2_ showed the highest performance, achieving near-complete VOC removal (>99%) across all chemical families, consistent with its strong solubilization capacity for both hydrophobic and polar compounds. Ethanol also provided high overall removal (94%), particularly for amines (98%) and terpenes (93%), reflecting its ability to solubilize a broad range of VOCs while remaining safer and more sustainable than CS_2_. Acetone showed more variable behavior: removal of aromatics and terpenes was high (98% and 97%, respectively), whereas aliphatic hydrocarbons were less effectively extracted (70%), suggesting a lower affinity for linear non-polar compounds.

Overall, Soxhlet extraction proved highly effective for removing both surface and deeply embedded VOCs. Among the tested solvents, ethanol offers the best compromise between efficiency, safety, and sustainability, whereas CS_2_, despite its superior performance, should be regarded primarily as a laboratory benchmark due to its toxicity and handling constraints. Therefore, safer alternatives such as ethanol-based extraction or, preferably, aqueous surfactant-assisted washing should be prioritized for scale-up. In this regard, SDS-assisted ultrasound and reflux washing provided high VOC removal efficiencies while avoiding the use of hazardous organic solvents, making them more suitable candidates for industrial deodorization of rHDPE.

### 3.3. Comparative Study of Mechanical and Physicochemical Properties of Post-Wash Recycled PE: Agitation, Ultrasonic, and Reflux Heating

**Figure 3:**
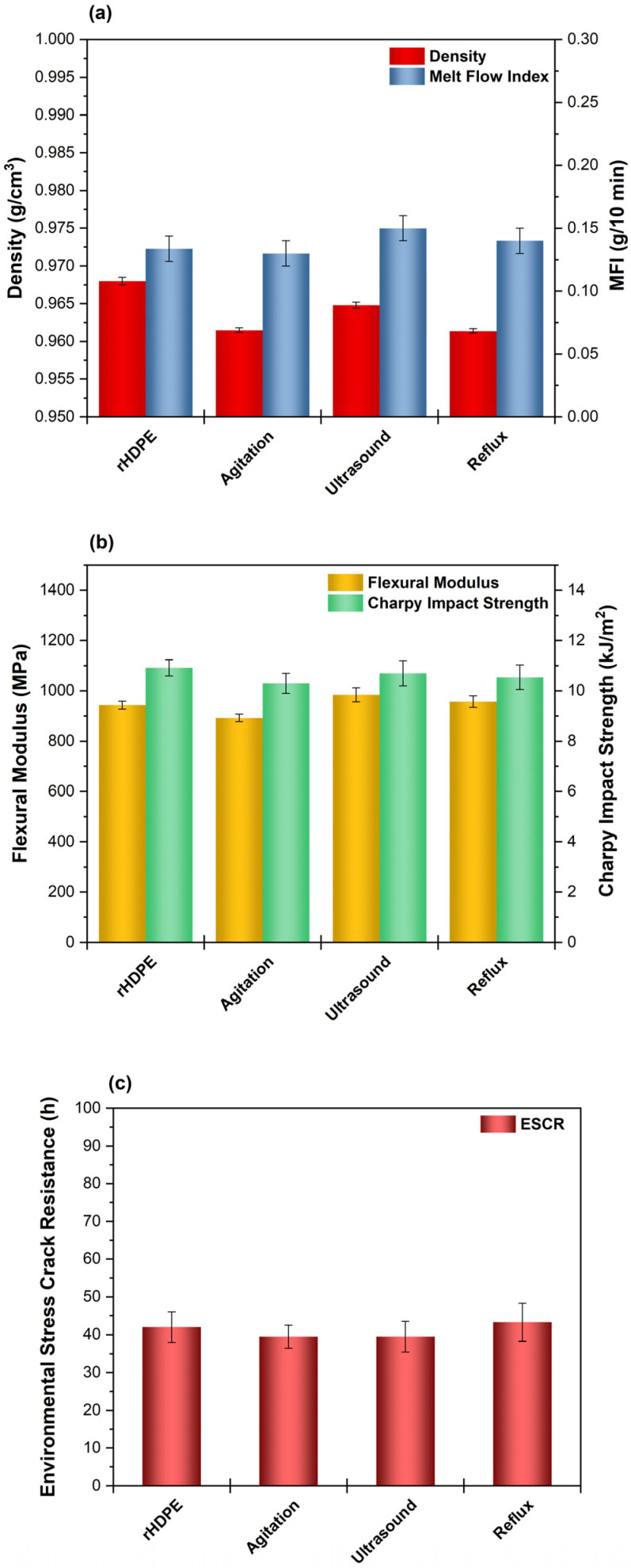
Mechanical and physicochemical properties of post-wash recycled PE: (**a**) Density and melt flow index, (**b**) flexural modulus and Charpy impact strength, and (**c**) ESCR resistance.

In addition to VOC removal, it is essential to evaluate the impact of each cleaning method on the physical and mechanical properties of recycled polyethylene (rHDPE). The ideal system should not only eliminate contaminants but also preserve or improve the material’s functional performance. The effectiveness of each treatment was evaluated based on four key parameters for the target application: density, melt flow index (MFI), mechanical properties, and environmental stress crack resistance (ESCR) [30]. Results are summarized in Figure 3. The Soxhlet extraction washing system was not included in this comparative analysis because, although it is very effective, its removal efficiency is not significantly different from that obtained with methods that have lower energy costs, such as the solvent reflux washing system, and to avoid the use of pure organic solvents instead of water.

The density of untreated rHDPE (0.968 g cm^−3^) remained unchanged after washing, with all treated samples falling within the typical range for HDPE (0.960–0.970 g cm^−3^; Figure 3a). Melt flow index showed only minor variations after treatment, with slightly higher values for ultrasound-treated samples, likely due to partial removal of low-molecular-weight species. Overall, no discernible indication of significant polymer degradation or loss of processability was observed based on the density and melt flow index measurements [31].

Analyzing the mechanical properties (Figure 3b) revealed no significant differences in flexural modulus or impact strength between untreated and washed rHDPE samples. Similarly, impact strength values remained stable, with values above 10 kJ/m^2^ and therefore within the range of container applications, including detergency. This result confirms that the material’s stiffness and impact resistance are preserved, indicating that VOC removal did not induce embrittlement or microstructural damage.

Finally, when analyzing the environmental stress cracking resistance, no significant improvement was observed after any of the washing treatments for the recycled material (Figure 3c). Despite the effective removal of VOCs, including potentially stress-concentrating species such as aromatic compounds and amines, the resistance to environmental cracking remained similar to that of the untreated sample. This outcome may be attributed to several factors. First, the inherent high crystallinity of rHDPE naturally makes it less resistant to stress cracking. Second, the removal of certain VOCs (especially amines) may have inadvertently reduced ductility, as some of these compounds could have acted as internal plasticizers. Their absence may increase matrix stiffness, favoring crack propagation under stress. These results indicate that ESCR is primarily governed by polymer structure rather than residual VOC content.

In summary, the washing processes do not compromise the properties of recycled PE, demonstrating that the treatments are mechanically safe. This ensures that the material maintains its integrity and performance, favoring its reuse in future applications in a more efficient manner and with higher recycling quality.

### 3.4. Removal of VOCs in Recycled PE by Dissolution/Precipitation Technique

In addition to solvent washing systems, a more advanced method based on dissolution and precipitation was tested to evaluate its ability to purify recycled polyethylene (rHDPE) beyond surface VOC removal. This procedure is based on the selective dissolution of the polymer in a suitable solvent and its subsequent precipitation by means of an antisolvent, which favors the separation of soluble contaminants in the liquid phase [32,33].

The treatment resulted in a clear separation between the purified polymer and a set of insoluble residues, which included not only VOCs, but also intentionally added substances (IAS) and non-intentionally added substances (NIASs), including inorganic compounds, present in the recycled material. The visual appearance of the recovered polymer differed noticeably from that of the untreated rHDPE. The purified material showed a lighter and more homogeneous coloration, suggesting that the process effectively removed not only VOCs but also pigment residues and solid contaminants that remain embedded in the recycled matrix after conventional washing (Figure S1).

**Figure 4:**
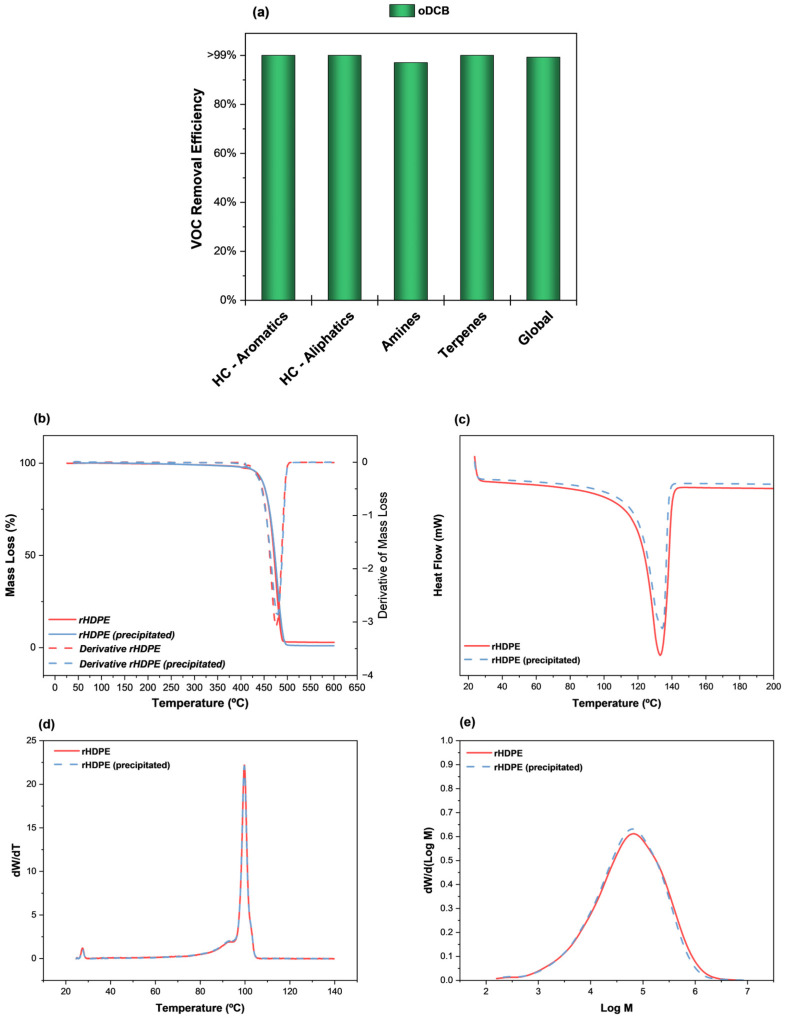
(**a**) VOC removal efficiency for dissolution/precipitation process and rHDPE and rHDPE precipitated results for (**b**) TGA; (**c**) DSC; (**d**) TREF; (**e**) GPC.

In terms of removal efficiencies (Figure 4a), aliphatic hydrocarbons, aromatics and terpenes show near complete removal of VOCs. Terpenes, which were one of the families that received the most attention due to their influence on odor, were also nearly completely removed below the detection limit of the equipment, followed by amines, with 97%. The complete removal of these compounds suggests that the oDCB dissolution system and subsequent precipitation are highly effective not only for apolar or low molecular complexity compounds, but also for polar and basic compounds such as amines. This highlights the versatility of the method for removing different types of volatile contaminants [33,34].

Figure S5 shows the chromatogram obtained by GC-MS analysis of the recycled PE sample after the dissolution/precipitation process. The chromatogram shows very low intensity peaks that have not been quantified. This significant decrease evidences the efficiency of the dissolution/precipitation process in the removal of volatile compounds. In addition, the absence of other peaks in the chromatogram indicates that most of the VOCs present in the original sample have been removed.

Compared to the other methods studied, this process offered the highest removal efficiency of VOCs, including compounds that are more difficult to extract through conventional washing techniques. This confirms the greater depth of purification achievable when the polymer is fully solubilized, allowing VOCs to diffuse completely into the solvent phase before being separated by precipitation. However, this process not only allows for the removal of impurities beyond the surface but could also generate modifications in the molecular structure of the material, such as changes in molecular weight distribution or crystallinity [35,36]. For this reason, a characterization of the PE precipitate was performed using various analytical techniques, such as thermogravimetric analysis (TGA), differential scanning calorimetry (DSC), temperature rise elution fractionation (TREF) and gel permeation chromatography (GPC) [37,38,39,40]. These techniques were selected to analyze in detail the thermal, structural, and molecular properties of the polymer after the purification process and thus to evaluate whether the treatment influenced the integrity and original characteristics of the material.

Thermogravimetric analysis (TGA) was used to determine the thermal stability of recycled PE by recording the mass loss as a function of temperature. This analysis is used to identify the presence of possible solvent residues or impurities that have not been completely removed during the washing process [41]. Figure 4b shows the comparative TGA analysis between the recycled polyethylene (recycled PE) sample and the precipitated recycled polyethylene (precipitated recycled PE) after the dissolution/precipitation process. In the case of precipitated PE, the thermal decomposition is very similar to that of PE, indicating that the process of removing volatile compounds has not affected the stability of the material. However, a decrease in the residue is observed for the precipitated PE, which indicates that some impurities have been eliminated during this process.

Differential scanning calorimetry (DSC) was used to study the thermal transitions of recycled PE, such as melting temperature and crystallinity. These properties will determine whether the dissolution and precipitation process altered the crystalline structure of the polymer in any way [42,43]. Figure 4c shows the results. Both samples, recycled PE and precipitated recycled PE, showed a largely overlapping melting behaviour, indicating that the dissolution and precipitation process has not significantly affected the melting point of the polyethylene, suggesting that the crystalline structure of the polymer has been maintained after treatment [44,45]. Thus, no significant differences are observed in the thermal parameters evaluated between both samples, concluding that the precipitation process has preserved the thermal integrity of the polyethylene.

Additionally, temperature rise elution fractionation (TREF) was used to analyze the distribution of recycled PE fractions according to their crystallinity. This technique allows knowing the distribution of the different polymeric fractions, which helps to evaluate the homogeneity and behavior of the purified polymer [46]. Figure 4d presents the results of the TREF analysis comparing recycled PE and precipitated recycled PE. The chromatograms of both recycled PE and precipitated recycled PE show a main crystallization peak at 100 °C, indicating that there was no significant change in crystallization temperature between the samples. As for the area under the peak, which is related to the amount of crystalline fraction in the sample, the values of recycled PE and precipitated recycled PE are very similar, being slightly higher in the case of precipitated recycled PE. On the other hand, the soluble fraction of PE was 1.9%, while, in the precipitated recycled PE, it was reduced to 1.5%. This indicates that the dissolution/precipitation process was able to slightly reduce the amount of amorphous or soluble fraction, which includes possible impurities present in the polymer. In conclusion, the results of the TREF analysis show that the dissolution/precipitation process did not negatively affect the crystalline structure of the polyethylene and agree well with the other results.

Finally, gel permeation chromatography (GPC) was used to determine the average molecular weight and its distribution. Figure 4e shows the GPC results to compare the molecular weight distributions of recycled PE and precipitated recycled PE. Regarding the average molecular weight (Mw), precipitated PE presents a slightly higher value (145,400 g/mol) compared to recycled PE (133,300 g/mol). Similarly, average molecular number (Mn) also experiences a slight increase in precipitated recycled PE (11.600 g/mol versus 11.300 g/mol in recycled PE). These results indicate that the dissolution/precipitation process did not generate significant degradation of the material. The dispersion index (Mw/Mn), which represents the width of the molecular weight distribution, is slightly higher in precipitated recycled PE (12.54) than in recycled PE (11.79). The intrinsic viscosity (IV) parameter shows an increase from 1.63 dL/g in recycled PE to 1.72 dL/g in precipitated recycled PE. This increase in intrinsic viscosity is consistent with the slight increase in average molecular weight and suggests that the precipitation process has not modified the molecular structure [47,48].

### 3.5. Techno-Economic Analysis

This techno-economic assessment aims to identify the deodorization technique with the highest technical and economic feasibility to be deployed on a market-scale plastic recycling plant. The five deodorization techniques were conceptually scaled up to a 20,000 tons/year HDPE recycling capacity. Aspen Plus^®^ v14 software was applied to simulate each deodorization process, providing energy and material consumption, and specifying proper unit operations corresponding to the explained experimental procedures. Having the solvent and utility consumption rates from the process simulation and equipment sizing calculation results, the capital cost and operating cost are estimated using standard cost models. Capital costs include capital for equipment, onsite and offsite infrastructure, design and engineering, and contingencies, while operating costs cover raw material, utilities, labor, and other indirect expenses.

To enable a consistent comparison between deodorization techniques that differ in both performance and cost structure, a techno-economic score was defined as the ratio of VOC removal efficiency to deodorization cost. This score is intended as a screening-level comparative indicator under consistent system boundaries and assumptions, rather than a comprehensive economic optimization metric. Its purpose is to support the relative ranking of alternative techniques by jointly considering deodorization effectiveness and cost:

$$Techno-economic\;Score=\frac{VOC\;Removal\;Efficiency\;(\%)}{Deodorization\;cost\;(€/kg)}\times 100$$

Higher values of the techno-economic score indicate greater feasibility, favoring deodorization techniques that combine low cost with high VOC removal efficiency.

**Figure 5:**
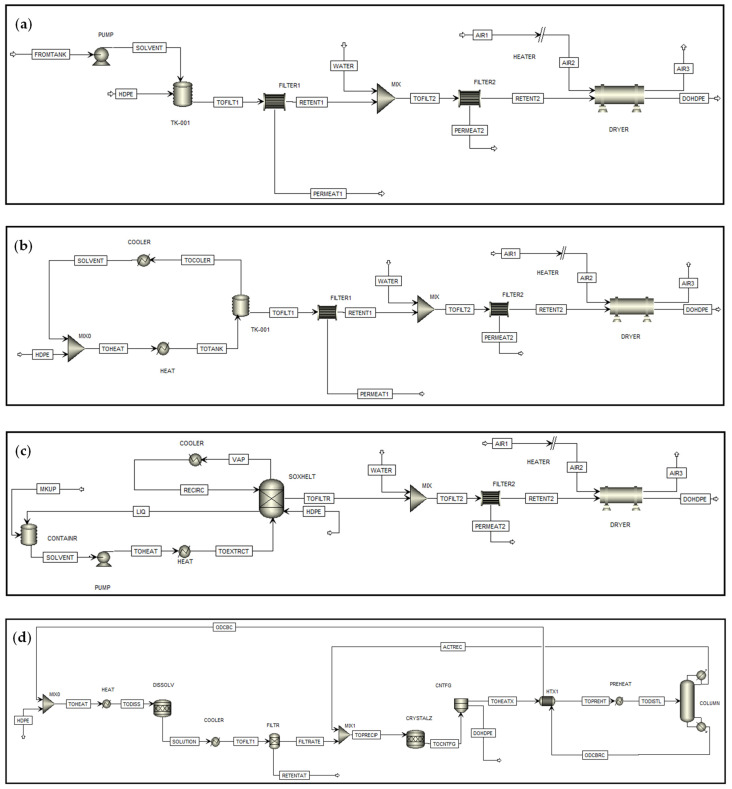
Aspen Plus simulation flow diagrams for different washing techniques: (**a**) agitation or ultrasound washing, (**b**) reflux heating, (**c**) Soxhlet extraction, and (**d**) dissolution/precipitation.

Simulations assume continuous plant operation with 95% annual availability, standard heat integration, and solvent recycling where applicable. Solvent reuse and spent-solvent management were considered in the techno-economic analysis. As detailed in the Supplementary Information, solvents were assumed to be reused for 150 washing batches before replacement, and the associated spent-solvent management cost was included in the operating cost calculation. Solvent recovery units, including condensers and distillation/heat recovery systems, were included where applicable. Flowsheet diagrams from Aspen Plus^®^, developed for different washing techniques, are presented in Figure 5. In the agitation and ultrasound washing processes (Figure 5a), recycled plastic particles are loaded into an agitated tank or ultrasonic bath, respectively, and mixed with solvent supplied by a loading pump. After the washing step, the mixture is transferred to a rotational disc filter to separate the solvent from the plastic, which is collected for reuse. The plastic particles are then rinsed with water in a second filtration step and finally conveyed to a continuous dryer to remove residual moisture. The reflux heating technique (Figure 5b) follows the same process sequence as agitation washing, but the washing step is conducted at an elevated temperature using an electrical heater to enhance solvent effectiveness. A water-cooled condenser is integrated into the system to recover any vaporized solvent and return it to the agitated tank.

The Soxhlet technique is simulated as a packed-bed extraction column filled with recycled plastic particles, assuming a 50% void fraction. The solvent circulates continuously through the bed in both liquid and vapor phases. A steam heater vaporizes the solvent before it enters the column, while a water-cooled condenser recovers the vaporized solvent for recycling. A circulation pump ensures a steady solvent flow throughout the process (Figure 5c).

The dissolution/precipitation process (Figure 5d) involves dissolving the plastic in a hot solvent within a heated agitation tank reactor, followed by solid-liquid separation to remove undissolved contaminants. The resulting polymer solution is then transferred to a precipitation tank, where acetone induces polymer precipitation. The precipitated plastic is separated using a centrifuge, while oDCB and acetone are recovered for reuse through a distillation column and heat recovery system.

A comprehensive description of the techno-economic analysis methodology, including system boundaries, cash-flow formulation, cost correlations, equipment sizing procedures, economic assumptions, raw material prices, and cost indices, is provided in the Supplementary Word File. In addition, detailed numerical results and intermediate cost calculations for all deodorization scenarios are reported in the accompanying Excel-based Supplementary Information. These materials ensure full transparency and reproducibility of the techno-economic assessment.

The results of the cost analysis (Table 1) show that most of the deodorization techniques have similar capital costs (~0.02–0.05 €/kg), except for dissolution-precipitation, which has a significantly higher capital cost (0.82 €/kg) due to its complex process. Residence time for washing equipment meaningfully affects the capital cost, such that a lower residence time requires smaller equipment and results in lower capital cost.

On the other hand, the operating costs show a wide range between different techniques, depending on the type of solvent, operating temperature, and operating time. Techniques using aqueous surfactant solutions (e.g., SDS, SDBS, CTAB in water) show the lowest operating costs (as low as 0.03 €/kg), due to lower surfactant powder consumption per weight of recycled HDPE. While liquid solvents such as ethanol, ethyl acetate, acetone and CS_2_ have significantly higher operating costs (up to 0.59 €/kg) due to the significant solvent volume consumption.

Higher operating temperatures for reflux heating and Soxhlet techniques tend to result in higher operating costs due to increased utility consumption. The dissolution/precipitation process, which requires the highest temperature and solvent volumes, has the highest total cost at 5.93 €/kg, making it impractical for industrial-scale application.

**Figure 6:**
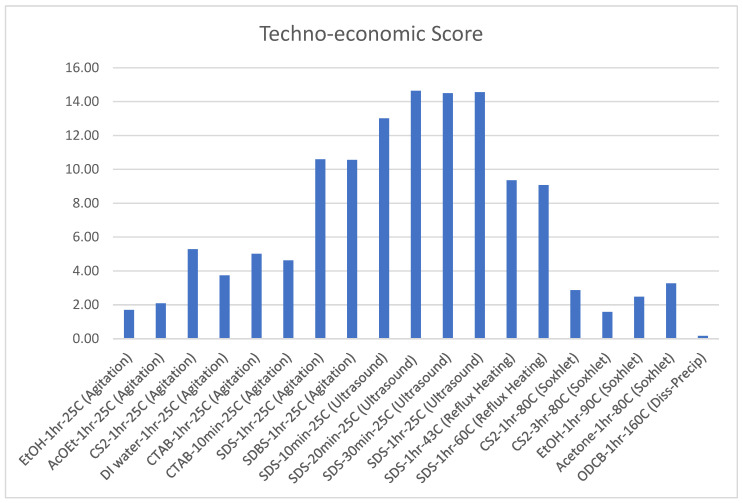
The techno-economic score for different washing techniques.

The techno-economic score (Figure 6), defined as the ratio of VOC removal efficiency to deodorization cost, highlights key performance differences. The ultrasonic washing technique using SDS at 25 °C achieves the highest score (13.01–15.21) across different washing durations (10–60 min), due to its combination of high VOC removal efficiency and low cost.

In contrast, while the dissolution/precipitation method provides one of the highest VOC removal efficiencies, its extremely high cost results in a very low techno-economic score; substantially higher costs under the investigated conditions limit its economic attractiveness in comparison to washing-based deodorization routes.

## 4. Conclusions

This study systematically compared different washing and purification methods to remove volatile organic compounds (VOCs) and non-intentionally added substances (NIASs) from post-consumer recycled high-density polyethylene (rHDPE). The evaluated techniques included agitation washing, ultrasound-assisted washing, reflux heating, Soxhlet extraction, and dissolution/precipitation.

All methods demonstrated efficacy in achieving substantial VOC removal without compromising the mechanical, thermal, or structural integrity of rHDPE. The following strategies were evaluated in this study:

Ultrasound-assisted washing with SDS and reflux-heated washing provided the best compromise between VOC removal efficiency and operational simplicity, whereas Soxhlet extraction and dissolution/precipitation achieved high efficiencies at the expense of energy demand, solvent use, and scalability constraints.

A techno-economic assessment identified ultrasound-assisted washing as the most cost-effective and scalable solution. This method combines high removal efficiency with operational simplicity, reduced energy consumption, and lower capital cost compared to solvent-intensive or dissolution-based alternatives.

The findings outlined herein provide a practical framework for implementing industrial decontamination of rHDPE, thereby facilitating its use in higher-value applications and contributing to the development of a more circular plastic economy.

## Figures and Tables

**Table 1:** Cost calculation results for recycled HDPE deodorization techniques.

WASHING TECHNIQUE	CAPITAL COSTS (€/KG)	OPERATING COST (€/KG)	DEODORIZATION COST (€/KG)
**ETOH-1HR-25 °C (AGITATION)**	0.03	0.17	0.20
**ACOET-1HR-25 °C (AGITATION)**	0.03	0.16	0.19
**CS2-1HR-25 °C (AGITATION)**	0.03	0.16	0.19
**DI WATER-1HR-25 °C (AGITATION)**	0.03	0.03	0.06
**CTAB-1HR-25 °C (AGITATION)**	0.03	0.04	0.07
**CTAB-10MIN-25 °C (AGITATION)**	0.03	0.04	0.06
**SDS-1HR-25 °C (AGITATION)**	0.03	0.03	0.06
**SDBS-1HR-25 °C (AGITATION)**	0.03	0.03	0.06
**SDS-10MIN-25 °C (ULTRASOUND)**	0.02	0.03	0.05
**SDS-20MIN-25 °C (ULTRASOUND)**	0.02	0.03	0.05
**SDS-30MIN-25 °C (ULTRASOUND)**	0.02	0.03	0.05
**SDS-1HR-25 °C (ULTRASOUND)**	0.03	0.04	0.07
**SDS-1HR-43 °C (REFLUX HEATING)**	0.03	0.05	0.08
**SDS-1HR-60 °C (REFLUX HEATING)**	0.03	0.08	0.11
**CS2-1HR-80 °C (SOXHLET)**	0.02	0.33	0.35
**CS2-3HR-80 °C (SOXHLET)**	0.05	0.59	0.63
**ETOH-1HR-90 °C (SOXHLET)**	0.02	0.36	0.38
**ACETONE-1HR-80 °C (SOXHLET)**	0.02	0.26	0.28
**ODCB-1HR-160 °C (DISS-PRECIP)**	0.82	5.11	5.93

## Data Availability

Data are contained within the article or Supplementary Material.

## Supplementary Materials

The following supporting information can be downloaded at: https://www.mdpi.com/article/10.3390/polym18121441/s1. Figure S1: Recycled HDPE before (left) and after (right) the dissolution process.; Table S1: Volatile organic compounds (VOCs) measured by GC-MS in recycled PE; Figure S2. GC-MS chromatogram of recycled PE; Figure S3. Percentage distribution of VOCs in recycled PE; Figure S4. Classification of VOCs by category; Figure S5. GC-MS chromatogram of the recycled PE precipitated sample; Table S2. Apparent polymer–wash distribution coefficients in ethanol for representative analytes; Table S3. Equipment cost correlation parameters; Table S4. The main techno-economic assumptions; Table S5. Raw materials unit price [49,50,51,52,53,54,55,56].

## References

[1] Plastics Europe Plastics—The Fast Facts 2024. https://plasticseurope.org/es/knowledge-hub/plastics-the-fast-facts-2024/.

[2] European Commission A European Strategy for Plastics in a Circular Economy. https://eur-lex.europa.eu/legal-content/EN/TXT/?qid=1516265440535&uri=COM:2018:28:FIN.

[3] Plastics Europe The Circular Economy for Plastics—A European Analysis 2024 • Plastics Europe. https://plasticseurope.org/knowledge-hub/the-circular-economy-for-plastics-a-european-analysis-2024/.

[4] Hodgson S.C., Casey R.J., Bigger S.W., Scheirs J. (2000). Review of Volatile Organic Compounds Derived from Polyethylene. Polym.—Plast. Technol. Eng..

[5] Maleknia S.D., Berger J., Odermatt J. (2016). Identification of Volatile Organic Compounds of Solid Waste by Pyrolysis GC/MS for Environmental Impact and Green Manufacturing. Air Qual. Clim. Change.

[6] De Somer T., Roosen M., Harinck L., Van Geem K.M., De Meester S. (2022). Removal of Volatile Components from Plastic Waste in Liquid Media: Effect of Temperature and Particle Size. Resour. Conserv. Recycl..

[7] Monti M., Perin E., Conterosito E., Romagnolli U., Muscato B., Girotto M., Scrivani M.T., Gianotti V. (2023). Development of an Advanced Extrusion Process for the Reduction of Volatile and Semi-Volatile Organic Compounds of Recycled HDPE from Fuel Tanks. Resour. Conserv. Recycl..

[8] Cherif Lahimer M., Ayed N., Horriche J., Belgaied S. (2017). Characterization of Plastic Packaging Additives: Food Contact, Stability and Toxicity. Arab. J. Chem..

[9] Paiva R., Wrona M., Nerín C., Bertochi Veroneze I., Gavril G.L., Andrea Cruz S. (2021). Importance of Profile of Volatile and Off-Odors Compounds from Different Recycled Polypropylene Used for Food Applications. Food Chem..

[10] Rung C., Welle F., Gruner A., Springer A., Steinmetz Z., Munoz K. (2023). Identification and Evaluation of (Non-)Intentionally Added Substances in Post-Consumer Recyclates and Their Toxicological Classification. Recycling.

[11] Even M., Hutzler C., Wilke O., Luch A. (2020). Emissions of Volatile Organic Compounds from Polymer-Based Consumer Products: Comparison of Three Emission Chamber Sizes. Indoor Air.

[12] Garofalo E., Taurino L., Di Maio L., Neitzert H.C., Incarnato L. (2023). Assessment of Melt Compounding with Zeolites as an Effective Deodorization Strategy for Mixed Plastic Wastes and Comparison with Degassing. Polymers.

[13] Wu S., Li D., Li H., Su Q.Z., Liang J., Zheng J., Zhong H.N., Dong B. (2024). Characterization and Elimination Efficiency of Volatile Organic Compounds in Mechanically Recycled Polyethylene Terephthalate at Various Recycling Stages. Waste Manag..

[14] Roosen M., Van Laere T., Decottignies V., Morel L., Schnitzler J.L., Schneider J., Schlummer M., Lase I.S., Dumoulin A., De Meester S. (2023). Tracing the Origin of VOCs in Post-Consumer Plastic Film Bales. Chemosphere.

[15] Wypych G. (2017). Methods of Odor Removal. Handbook of Odors in Plastic Materials.

[16] Dany H., Dhong W.W., Jiat K.W., Leong T.K., Yuhana N.Y., Tan G. (2021). Deodorizing Methods for Recycled High-Density Polyethylene Plastic Wastes. Mater. Plast..

[17] Kol R., Roosen M., Ügdüler S., Van Geem K.M., Ragaert K., Achilias D.S., De Meester S., Kol R., Roosen M., Ügdüler S. (2021). Recent Advances in Pre-Treatment of Plastic Packaging Waste. Waste Material Recycling in the Circular Economy—Challenges and Developments.

[18] Fuller J., White D., Yi H., Colley J., Vickery Z., Liu S. (2020). Analysis of Volatile Compounds Causing Undesirable Odors in a Polypropylene—High-Density Polyethylene Recycled Plastic Resin with Solid-Phase Microextraction. Chemosphere.

[19] Roosen M., Harinck L., Ügdüler S., De Somer T., Hucks A.G., Belé T.G.A., Buettner A., Ragaert K., Van Geem K.M., Dumoulin A. (2022). Deodorization of Post-Consumer Plastic Waste Fractions: A Comparison of Different Washing Media. Sci. Total Environ..

[20] Ügdüler S., Van Geem K.M., Roosen M., Delbeke E.I.P., De Meester S. (2020). Challenges and Opportunities of Solvent-Based Additive Extraction Methods for Plastic Recycling. Waste Manag..

[21] Chen Z.F., Lin Q.B., Su Q.Z., Zhong H.N., Nerin C. (2021). Identification of Recycled Polyethylene and Virgin Polyethylene Based on Untargeted Migrants. Food Packag. Shelf Life.

[22] Xu Z., Sanchez-Rivera K., Granger C., Zhou P., del Carmen Munguia-Lopez A., Ikegwu U.M., Avraamidou S., Zavala V.M., Van Lehn R.C., Bar-Ziv E. (2025). Solvent-Based Plastic Recycling Technologies. Nat. Chem. Eng..

[23] Blanco A., Juan R., Istrate R., Paredes B., Martin-Gamboa M., Domínguez C., Dufour J., García-Muñoz R.A. (2024). Assessing the Circularity of Post-Consumer HDPE Milk Bottles through Open-Loop Recycling and Their Environmental Impact. Clean. Environ. Syst..

[24] Cabanes A., Valdés F.J., Fullana A. (2020). A Review on VOCs from Recycled Plastics. Sustain. Mater. Technol..

[25] Roosen M., De Somer T., Demets R., Ügdüler S., Meesseman V., Van Gorp B., Ragaert K., Van Geem K.M., Walgraeve C., Dumoulin A. (2021). Towards a Better Understanding of Odor Removal from Post-Consumer Plastic Film Waste: A Kinetic Study on Deodorization Efficiencies with Different Washing Media. Waste Manag..

[26] Bruce C.D., Berkowitz M.L., Perera L., Forbes M.D.E. (2002). Molecular Dynamics Simulation of Sodium Dodecyl Sulfate Micelle in Water: Micellar Structural Characteristics and Counterion Distribution. J. Phys. Chem. B.

[27] Trujillo C., Sánchez-Sanz G. (2016). A Study of π-π Stacking Interactions and Aromaticity in Polycyclic Aromatic Hydrocarbon/Nucleobase Complexes. ChemPhysChem.

[28] Ferreira A.M., Sucena I., Otero V., Angelin E.M., Melo M.J., Coutinho J.A.P. (2022). Pretreatment of Plastic Waste: Removal of Colorants from Hdpe Using Biosolvents. Molecules.

[29] Vilaplana F., Karlsson S. (2008). Quality Concepts for the Improved Use of Recycled Polymeric Materials: A Review. Macromol. Mater. Eng..

[30] Juan R., Domínguez C., Robledo N., Paredes B., Galera S., García-Muñoz R.A. (2021). Challenges and Opportunities for Recycled Polyethylene Fishing Nets: Towards a Circular Economy. Polymers.

[31] Tan C.J., Andriyana A., Ang B.C., Wong D. (2020). Mechanical Deformation and Fracture Mechanisms of Polymeric Fibres from the Perspective of Fractography—A Review. Eur. Polym. J..

[32] Ezquerro Ó., Pons B., Tena M.T. (2003). Evaluation of Multiple Solid-Phase Microextraction as a Technique to Remove the Matrix Effect in Packaging Analysis for Determination of Volatile Organic Compounds. J. Chromatogr. A.

[33] Poulakis J.G., Papaspyrides C.D. (1995). Dissolution/Reprecipitation Technique Applied on High-Density Polyethylene: I. Model Recycling Experiments. Adv. Polym. Technol..

[34] Achilias D.S., Giannoulis A., Papageorgiou G.Z. (2009). Recycling of Polymers from Plastic Packaging Materials Using the Dissolution-Reprecipitation Technique. Polym. Bull..

[35] Cohen S., Chejanovsky I., Suckeveriene R.Y. (2022). Grafting of Poly(Ethylene Imine) to Silica Nanoparticles for Odor Removal from Recycled Materials. Nanomaterials.

[36] Patel S.H., Xanthos M. (2001). Environmental Issues in Polymer Processing: A Review on Volatile Emissions and Material/Energy Recovery Options. Adv. Polym. Technol..

[37] Chrysafi I., Asimakidou T., Michailidou G., Xanthopoulou E., Tziamtzi C.K., Zamboulis A., Bikiaris D.N. (2022). Characterization of the Thermal, Structural, and Mechanical Properties of Recycled HDPE. Macromol. Symp..

[38] Monrabal B., Lõpez E., Romero L. (2013). Advances in Thermal Gradient Interaction Chromatography and Crystallization Techniques for Composition Analysis in Polyolefins. Macromol. Symp..

[39] Juan R., Paredes B., García-Muñoz R.A., Domínguez C. (2021). Quantification of PP Contamination in Recycled PE by TREF Analysis for Improved the Quality and Circularity of Plastics. Polym. Test..

[40] Blanco A., Juan R., Paredes B., Domínguez C., García-Muñoz R.A. (2025). Advancing Polypropylene Quantification in Recycled HDPE: A Comparative Study of FTIR, DSC, TREF, and TGIC for Enhanced Plastic Circularity. Polym. Test..

[41] Menczel J.D., Prime R.B. (2008). Thermal Analysis of Polymers: Fundamentals and Applications.

[42] Kong Y., Hay J.N. (2002). The Measurement of the Crystallinity of Polymers by DSC. Polymer.

[43] Khan S., Uz Zaman A., Junaid M., Shehbaz T., Awais Khan M., Nawaz Khan F., Ramzan Abdul Karim M. (2023). Effect of Mass and Temperature Rate on the Thermal Properties of High-Density Polyethylene Using Differential Scanning Calorimetry. Digit. Manuf. Technol..

[44] Wang G., Harrison I.R. (1994). Polymer Melting: Heating Rate Effects on DSC Melting Peaks. Thermochim. Acta.

[45] Wong A.C.Y., Lam F. (2002). Study of Selected Thermal Characteristics of Polypropylene/Polyethylene Binary Blends Using DSC and TGA. Polym. Test..

[46] Monrabal B. (2006). Temperature Rising Elution Fractionation and Crystallization Analysis Fractionation. Encyclopedia of Analytical Chemistry.

[47] Arndt J.H., Macko T., Vanderfeesten J., Verhoogt H., Brüll R. (2024). Characterizing Graft Distribution in Maleic Anhydride Grafted Polyethylene—GPC with IR and UV-Detection. J. Chromatogr. A.

[48] Hintersteiner I., Himmelsbach M., Buchberger W.W. (2015). Characterization and Quantitation of Polyolefin Microplastics in Personal-Care Products Using High-Temperature Gel-Permeation Chromatography. Anal. Bioanal. Chem..

[49] Sinnott R., Towler G. (2020). Chapter 6—Costing and Project Evaluation. Chemical Engineering Design.

[50] Product Image Search on Alibaba. https://www.alibaba.com/trade/search?spm=a2700.details.0.0.10f84a6dO0mr2C&scene=invalid_items_initiate_imgsrch&escapeQp=true&IndexArea=image_similar&productId=1600530269065.

[51] Chemical Process Design and Integration, 2nd Edition|Wiley, Wiley.com. https://www.wiley.com/en-us/Chemical+Process+Design+and+Integration%2C+2nd+Edition-p-9781118699089.

[52] Jierui Large Scale Single Tank 2500l Ultrasonic Cleaner Machine. https://www.alibaba.com/product-detail/Jierui-Large-Scale-Single-Tank-2500L_1600562398935.html?spm=a2700.galleryofferlist.p_offer.d_title.2db713a0aAFLvT&s=p.

[53] Intratec|Reliable & Independent Information about Commodities. https://www.intratec.us/.

[54] IndexBox—Plataforma de Inteligencia de Mercado—Datos, Herramientas y Análisis. https://es.indexbox.io/.

[55] Global Chemical and Petrochemicals, Specialty Chemicals, Elastomer and Rubber, Fertilizer and Feedstock—Latest Chemical Prices, News and Market Analysis|ChemAnalyst. https://www.chemanalyst.com/.

[56] Find quality Manufacturers, Suppliers, Exporters, Importers, Buyers, Wholesalers, Products and Trade Leads from Our Award-Winning International Trade Site. Import & Export on alibaba.com. Alibaba. https://www.alibaba.com.

