# Supplementary material for: Deodorization of Recycled HDPE: Comparative Assessment of Washing and Solvent-Based Purification Strategies with a Techno-Economic Analysis
Source: Polymers (Basel). 2026 Jun 9;18(12):1441. doi: 10.3390/polym18121441 (PMC13306681; doi:10.3390/polym18121441)
Supplement: Supplementary file 1 [file polymers-18-01441-s001.zip › polymers-4304533-supplementary.pdf]

Supplementary Material

Deodorization of Recycled HDPE: Comparative Assessment of Washing and Solvent-Based Purification Strategies with a Techno-Economic Analysis

Aymara Blanco [1,2], Vafa Feyzi [3], Rafael Juan [1,2], Beatriz Paredes [1,2], Carlos Domínguez [1,2], Javier Dufour [2,3] and Rafael A. García-Muñoz [1,2,4,*]

1 Polymer Technology Laboratory (LATEP), Rey Juan Carlos University, Tulipán St., 28933 Móstoles, Madrid, Spain; aymara.blanco@urjc.es (A.B.); rafael.juan@urjc.es (R.J.); beatriz.paredes@urjc.es (B.P.); carlos.domiguez@urjc.es (C.D.)
2 GIQA, Group of Environmental and Chemical Engineering, ESCET, Rey Juan Carlos University, Tulipán St., 28933 Móstoles, Madrid, Spain; javier.dufour@imdea.org
3 Systems Analysis Unit, IMDEA Energy, Avda. Ramón de la Sagra 3, 28935 Móstoles, Madrid, Spain; vafa.feyzi@imdea.org
4 Instituto de Investigación de Tecnologías para la Sostenibilidad, Rey Juan Carlos University, Tulipán St., 28933 Móstoles, Madrid, Spain
* Correspondence: rafael.garcia@urjc.es

Table of contents

S1 Supplemental Results 2
S2 Apparent Polymer-Wash Distribution Coefficients in Ethanol 5
S3 Techno-Economic Analysis Methodology 6
References 8

S1 Supplemental Results

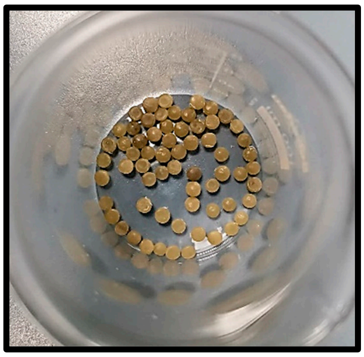

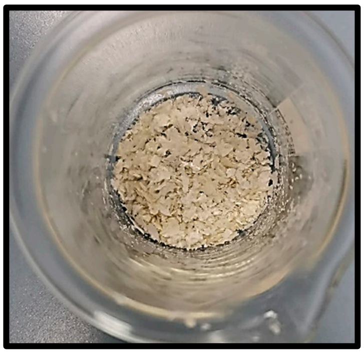

Figure S1. Recycled HDPE before (left) and after (right) the dissolution process.

Table S1. Volatile organic compounds (VOCs) measured by GC-MS in recycled PE.

Retention Time (min)	Compound	Area
1,813	Toluene	5,1E+06
2,056	Tetrachloroethylene	1,7E+06
2,501	Ethylbenzene	3,8E+06
2,572	1,3-dimethylbenzene	1,0E+07
2,734	p-Xylene	8,9E+06
2,831	o-Xylene	7,1E+06
3,679	1-Ethyl-4-methylbenzene	2,5E+06
3,772	1-Ethyl-3-methylbenzene	3,5E+06
3,790	1,2,4-trimethylbenzene	2,6E+06
4,245	Mesitylene	4,8E+06
4,304	Dean	1,5E+06
4,320	2,2,4,6,6-pentamethylheptane	1,7E+06
4,836	2-ethyl-1-hexanol	5,2E+06
4,958	d-Limonene	2,6E+06
5,549	2-Ethyl-1,4-dimethylbenzene	1,1E+06
6,204	2-Ethyl-1,3-dimethylbenzene	7,1E+05
6,543	Tridecane	1,1E+06
6,654	O-decylohydroxylamine	9,8E+05
6,656	Undecane	1,2E+06
6,976	1,2,3,4-tetramethylbenzene	7,3E+05
7,062	1,2,4,5-tetramethylbenzene	7,1E+05
7,640	2,2,6,6-tetramethyl-4-piperidinol	6,8E+05
9,170	Hexadecane	6,4E+05
9,257	4-methyl-1-undecene	1,8E+05
11,866	Isobutyl and nonyl oxalate	2,6E+05
14,506	Heptadecane	1,6E+05
14,522	Tetradecane	1,8E+05
17,088	2,4-Di-tert-butylphenol	5,2E+05
24,898	Dimethyl palmitamine	4,7E+06
24,900	2-[(4-bromo-1,1-diphenylethyl)oxy]ethyla-mine	1,5E+06
24,901	N,N-Dimethyloctylamine	3,8E+05

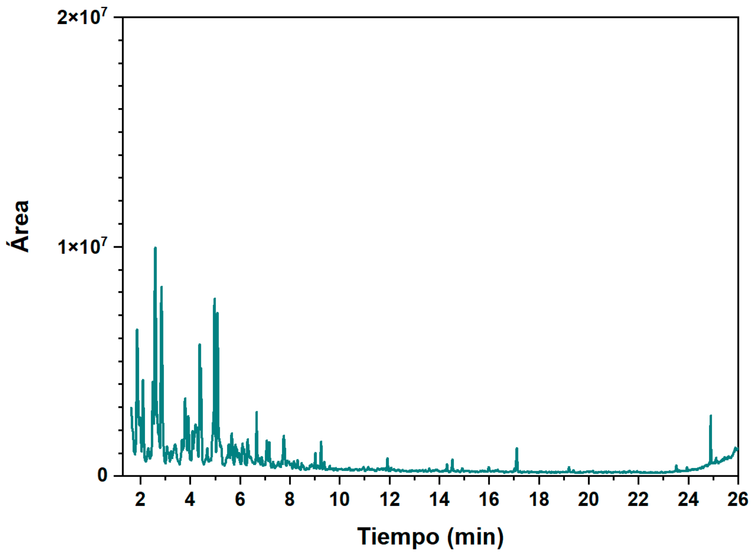

Figure S2. GC-MS chromatogram of recycled PE.

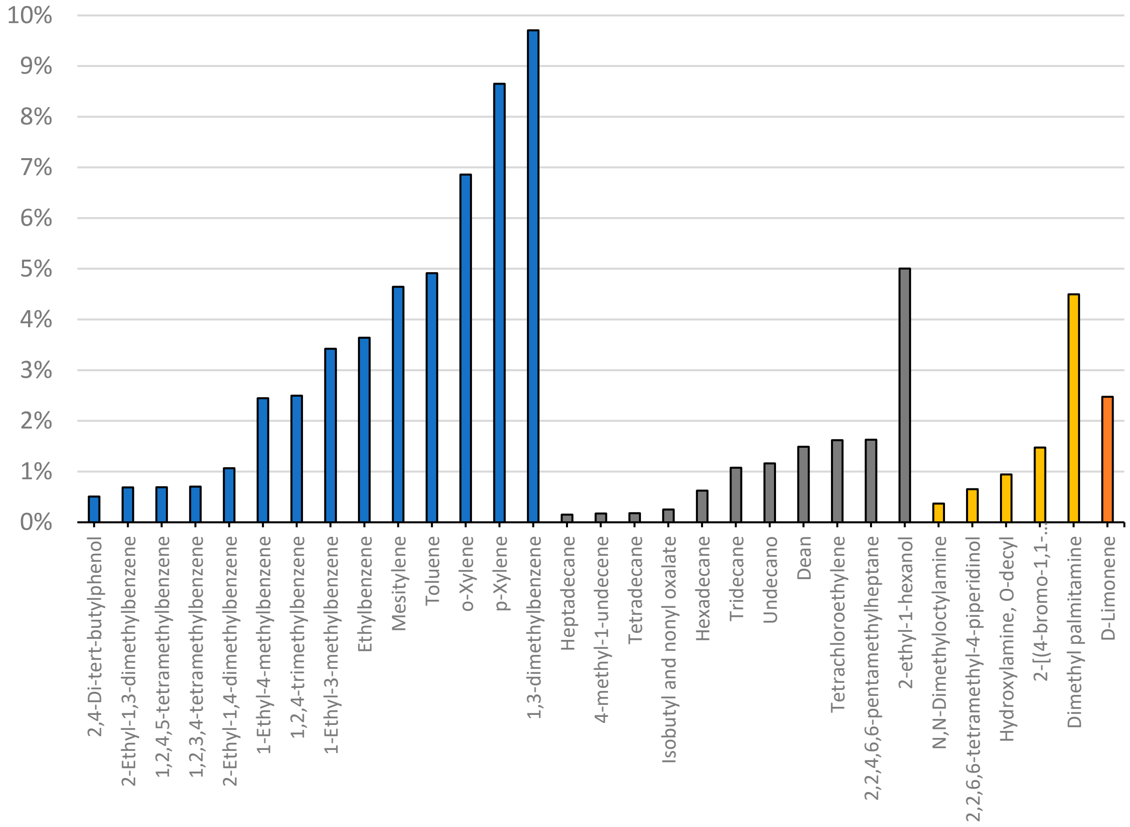

Figure S3. Percentage distribution of VOCs in recycled PE.

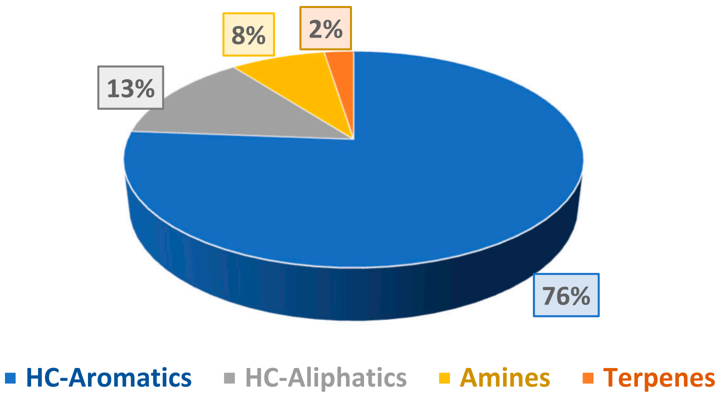

Figure S4. Classification of VOCs by category.

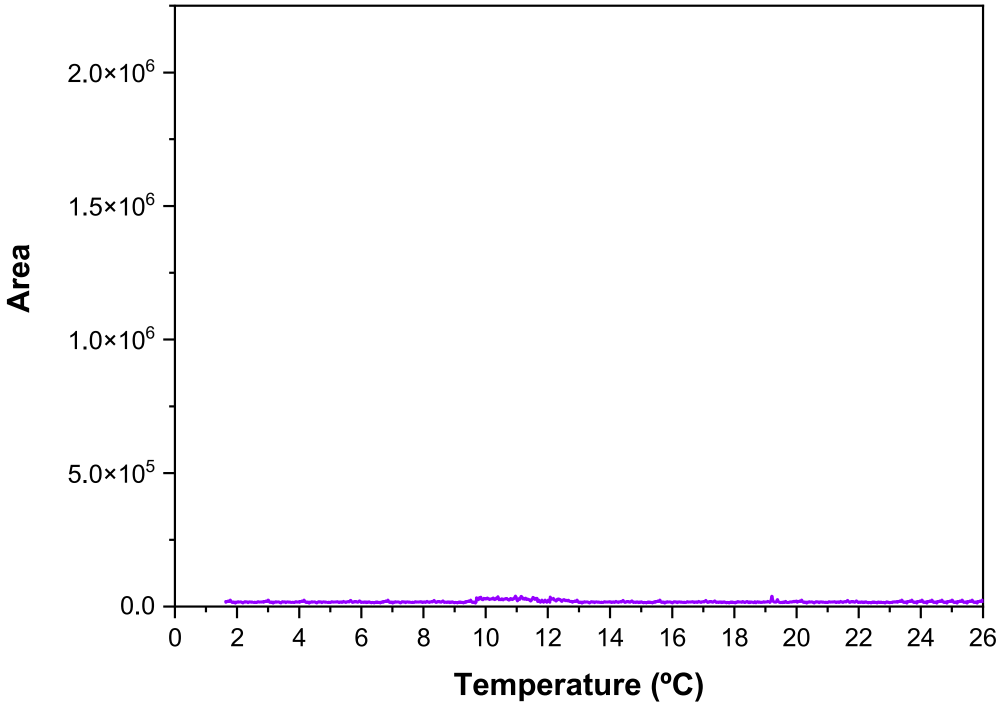

Figure S5. GC-MS chromatogram of the recycled PE precipitated sample.

S2 Apparent Polymer-Wash Distribution Coefficients In Ethanol

To provide additional mechanistic support for the discussion of solvent-polymer interactions, preliminary equilibrium screening tests were performed in ethanol using representative model compounds. These experiments were designed as screening measurements to estimate apparent polymer-wash distribution coefficients for selected analytes under sealed conditions.

Ethanol was selected because it provided the most stable and reproducible headspace GC/MS quantification under the analytical conditions used in this work. Equivalent determinations in aqueous and surfactant-containing media were not included, since matrix effects under the applied GC/MS protocol hindered robust and reproducible quantification.

The apparent polymer-wash distribution coefficient was calculated from a liquid-phase mass balance as:

$$m_{abs} = (C_0 - C_w^{eq})\ V$$

$$C_p^{eq} = m_{abs} / m_p$$

$$K^{app}_{p/w} = C_p^{eq} / C_w^{eq}$$

where m_{abs} is the mass of analyte apparently transferred from the liquid phase to the polymer, C_0 is the initial analyte concentration in the ethanol blank, C_w^{eq} is the equilibrium concentration in the ethanol phase after contact with PE, V is the ethanol volume, and m_p is the polymer mass. In these experiments, m_p = 0.18221 g and V = 1.0 mL.

For dilute ethanol solutions, concentrations reported in ppm were treated as approximately equivalent to μg mL^{-1}. Accordingly, C_p^{eq} is expressed in μg g^{-1} and $K^{app}_{p/w}$ in mL g^{-1}. These coefficients are reported as apparent values because they were derived from liquid-phase mass balance under simplified equilibrium screening conditions and were not corrected for possible headspace partitioning or direct measurement of analyte content in the polymer phase. Overall, these apparent coefficients are intended to provide qualitative mechanistic support for the discussion of solvent-polymer interactions rather than a complete thermodynamic description of all washing media. Their interpretation should therefore remain limited to the selected model analytes and the specific ethanol-based screening conditions used here.

Table S2. Apparent polymer–wash distribution coefficients in ethanol for representative analytes.

Analyte	C_0 (μg mL^{-1})	C_w^{eq} with PE (μg mL^{-1})	$\Delta C = C_0 - C_w^{eq}$ (μg mL^{-1})	C_p^{eq} (μg g^{-1})	$K^{app}_{p/w}$ (mL g^{-1})
Toluene	20.0	19.4	0.6	3.29	0.170
Hexane	20.0	11.5	8.5	46.65	4.06
Limonene	20.0	19.1	0.9	4.94	0.259
Ethylenediamine	1000.0	984.4	15.6	85.62	0.0870

Note. The relative standard deviation associated with polymer mass was 4.38% (0.18221 ± 0.00798 g), which represents the minimum propagated uncertainty in C_p^{eq} and $K^{app}_{p/w}$, excluding analytical uncertainty from GC/MS quantification.

S3 Techno-Economic Analysis Methodology

Techno-economic analysis was conducted to estimate the recycled HDPE deodorization cost and identify the primary cost drivers impacting the economic feasibility of different washing techniques. The operation of plastic deodorization processes was simulated in a large-scale scenario with its corresponding technical parameters. Process simulation and equipment sizing calculation results provide the data inventory needed for the techno-economic analysis.

The cost analysis methodology was developed according to the system design for an annual capacity of 20,000 tons of recycled plastic. The system boundary includes unit operations for batch-wise washing steps, solid-liquid separation, drying, and solvent recovery. Continuous flow rate of feeds and products, in addition to periodical replacement of consumables, determines the economic viability of each technique for recycled plastic deodorization. Considering the functional unit as treatment, of 1 kg of recycled HDPE, this cost assessment results in the deodorization cost as the economic indicator for techno-economic benchmarking and feasibility study.

The plastic deodorization cost was calculated through a cash flow analysis over the plant's lifespan, considering both fixed and operating costs. Fixed capital investment costs include inside battery limits, outside battery limits, design and engineering, and contingency costs. Inside battery limit cost items are estimated by following the factorial method, which calculates the total fixed cost of construction as factors of purchased equipment costs [1]. The primary capital equipment includes agitation tanks, ultrasonic bath, loading pump, dryer, centrifugal filter, heat exchangers, dissolution and precipitation reactors, and extraction and distillation columns. The total capital cost is annualized over the system's lifetime and discounted according to the following equation:

$$Annualized\ Capital\ Cost = Total\ Capital\ Cost \times \frac{i.(1+i)^n}{(1+i)^n - 1}$$

The equipment purchased cost is determined based on the equipment capacity (obtained from process simulation and equipment sizing), expressed as a power law of capacity:

$$C_E = C_B \times (\frac{Q}{Q_B})^M \times \frac{INDEX_1}{INDEX_2}$$

Where, C_E is the equipment cost with capacity Q, C_B is known based cost for equipment with capacity Q_B, and M is a constant depending on equipment type. As the existing equipment cost data is often old, such data is brought up to date and put on a 2025 price by multiplying by the ratio of cost indexes. Table S3. includes the parameters for the calculation of the equipment cost.

Table S3. Equipment cost correlation parameters.

Equipment	Min Scale (Q_B)	Unit	Max Scale	C_B	E_B	M	Currency	Ref
Centrifuge	1500	Lit/h	8000	11500	1500	0.75	USD-2025	[2]
Agitator	5	Kw	75	15000	990	1.05	USD-2007	[1]
Dryer	700	kg H_2O/hr	3000	230000	700	0.65	USD-2000	[3]
Ultrasonic Bath	2500	lit	2500	6980	2500	1	USD-2025	[4]
Extracting Column	0.1	m^3	20	4920	0.1	0.53	USD-2000	[3]
S&T Heat Exchanger	80	m^2	4000	32800	80	0.68	USD-2000	[3]
Centrifugal Pump	1	KW	10	1970	1	0.35	USD-2000	[3]
Vacuum Filter	10	m^2	180	96621	10	0.41	USD-2007	[1]

The main operational costs include solvent consumption, electricity and utility costs, and labor costs. Spent solvent management and wastewater treatment were included as operating cost items in the techno-economic analysis. Solvents (acetone, ethanol, ethyl

acetate, and carbon disulfide) were assumed to have a finite lifetime of 150 washing batches, after which an equivalent fraction of the circulating solvent inventory was replaced and treated off-site. Aqueous effluents generated during washing and rinsing steps were considered wastewater streams requiring treatment before discharge. Associated solvent disposal/recovery and wastewater treatment costs were included in the operating cost of each deodorization technique. Some of the main economic parameters considered in the techno-economic analysis are reported in Table S4.

Table S4. The main techno-economic assumptions.

Parameters	Value	Unit
Plant Capacity	20000	Tone HDPE/year
Plant lifetime	20	years
Solvents lifetime	150	Batches of washing
Interest rate	8	%
Electricity price	0.06	€/kWh
Steam price	30	€/tone
Cooling water	0.006	€/m^3 water in circulation
Wasted water treatment cost	0.5	€/m^3
Spent solvent disposal cost (Acetone/Ethanol/Ethyl acetate)	0.65	€/litter
Spent solvent disposal cost (CS_2)	1.5	€/litter
Worker's annual salary	30000	€/year
Supervision cost	25	% of labor cost
Equipment erection cost (ER)	20	% of total equipment cost
Piping cost (PP)	10	% of total equipment cost
Instrument and control cost (IC)	30	% of total equipment cost
Electrical equipment cost (EL)	20	% of total equipment cost
Civil works cost (CV)	30	% of total equipment cost
Structure and building cost (SB)	20	% of total equipment cost
Inside battery limit cost (ISBL)	ER+PP+IC+EL+CV+SB	-
Offsite battery limit (OSBL)	30	% of ISBL
Design and engineering cost	30	% of ISBL+OSBL
Contingency cost	10	% of ISBL+OSBL

The material cost includes periodic replacement of solvent and other raw materials needed to keep the continuous operation of the deodorization process. The unit price for these consumable materials is considered from the large-scale quantity purchasing price as reported in Table S5.

Table S5. Raw materials unit price.

Items	Value	Unit	Year	Source
DI water	2.22	€/ton	2026	[1]
Ethanol	1.07	€/kg	2026	[5]
Ethyl Acetate	1.40	€/kg	2026	[6]
Acetone	1.00	€/kg	2026	[6]
Carbon Disulfide	0.97	€/kg	Sep-24	[7]
Ortho-Dichlorobenzene (oDCb)	4.65	€/kg	2026	[8]
Cetyltrimethylammonium Bromide (CTAB)	40.00	€/kg	2026	[8]
Sodium Dodecyl Sulfate (SDS)	15.00	€/kg	2026	[8]
Sodium Dodecylbenzene Sulfonate (SDBS)	8.00	€/kg	2026	[8]

Cost calculations were performed using the euro currency in 2026 and considering Spain as the geographical region for installing the recycled HDPE deodorization plants.

References

1. R. Sinnott and G. Towler, “Chapter 6 - Costing and Project Evaluation,” in *Chemical Engineering Design (Sixth Edition)*, R. Sinnott and G. Towler, Eds., in Chemical Engineering Series. , Butterworth-Heinemann, 2020, pp. 275–369. doi: 10.1016/B978-0-08-102599-4.00006-0.
2. “Product image search on Alibaba.” Accessed: Jul. 24, 2025. [Online]. Available: https://www.alibaba.com/trade/search?spm=a2700.details.0.0.10f84a6dO0mr2C&scene=invalid_items_initiate_imgsrch&es-capeQp=true&IndexArea=image_similar&productId=1600530269065
3. “Chemical Process Design and Integration, 2nd Edition | Wiley,” Wiley.com. Accessed: Jul. 24, 2025. [Online]. Available: https://www.wiley.com/en-us/Chemical+Process+Design+and+Integration%2C+2nd+Edition-p-9781118699089
4. “Jierui Large Scale Single Tank 2500l Ultrasonic Cleaner Machine.” Accessed: Jul. 24, 2025. [Online]. Available: https://www.alibaba.com/product-detail/Jierui-Large-Scale-Single-Tank-2500L_1600562398935.html?spm=a2700.gallery-offerlist.p_offer.d_title.2db713a0aAFLvT&s=p
5. “Intratec | Reliable & Independent Information about Commodities.” Accessed: Jul. 24, 2025. [Online]. Available: https://www.intratec.us/
6. “IndexBox - Plataforma de inteligencia de mercado - Datos, Herramientas y Análisis.” Accessed: Jul. 24, 2025. [Online]. Available: https://es.indexbox.io/
7. “Global Chemical and Petrochemicals, Specialty Chemicals, Elastomer and Rubber, Fertilizer and Feedstock - Latest Chemical Prices, News and Market Analysis | ChemAnalyst.” Accessed: Jul. 24, 2025. [Online]. Available: https://www.chemanalyst.com/
8. “Find quality Manufacturers, Suppliers, Exporters, Importers, Buyers, Wholesalers, Products and Trade Leads from our award-winning International Trade Site. Import & Export on alibaba.com,” Alibaba. Accessed: Jul. 24, 2025. [Online]. Available: https://www.alibaba.com
